# Exploring the Interplay between Tribocorrosion and Surface Chemistry of the ASTM F139 Surgical Stainless Steel in Phosphate-Buffered Saline Solution

**DOI:** 10.3390/ma17102295

**Published:** 2024-05-13

**Authors:** Marcelo de Matos Macedo, Marcela Bergamaschi Tercini, Renato Altobelli Antunes, Mara Cristina Lopes de Oliveira

**Affiliations:** Centro de Engenharia, Modelagem e Ciências Sociais Aplicadas (CECS), Universidade Federal do ABC (UFABC), Santo André 09210-580, SP, Brazil; marcelo.macedo@ufabc.edu.br (M.d.M.M.); marcela.tercini@ufabc.edu.br (M.B.T.); renato.antunes@ufabc.edu.br (R.A.A.)

**Keywords:** ASTM F139 stainless steel, tribocorrosion, PBS, XPS, surface chemistry, passive film composition

## Abstract

Surgical ASTM F139 stainless steel is used for temporary fixtures in the biomedical field. Tribocorrosion is a major concern in this application. The aim of the present work was to study the interplay between tribocorrosion behavior and the surface chemistry of the ASTM F139 stainless steel in phosphate-buffered saline solution (PBS). Sliding wear tests were conducted against alumina balls at different electrochemical potentials: open circuit potential (OCP), cathodic potential (−100 mV versus the OCP), and anodic potentials (+200 mV_Ag/AgCl_ and +700 mV_Ag/AgCl_). The normal load was 20 N. The wear volume was estimated based on micrographs obtained from the wear tracks using confocal laser scanning microscopy. Moreover, the wear tracks were also examined by scanning electron microscopy (SEM). The surface chemistry of the ASTM F139 specimens was analyzed by X-ray photoelectron spectroscopy (XPS). The wear volume was dependent on the electrochemical potential, being maximized at +700 mVAg/AgCl. Delamination areas and grooves were observed in the wear tracks. Detailed assessment of the surface chemistry inside the wear tracks allowed identification of the main chemical species and their relative quantities, thus enabling correlation of the passive film composition with the observed tribocorrosion behavior.

## 1. Introduction

Austenitic stainless steels are the most common materials for temporary implant devices used for fracture healing, such as plates, screws, nails, and rods [1,2], as well as for hip joints, with a market share of around 70% in the USA [3]. This dominating usage arises from favorable features, such as ease of manufacturing at a relatively low cost, suitable mechanical properties, and biocompatibility [4,5]. ASTM F138 and ASTM F139 are the designations of implant-quality stainless steels for orthopedic applications [6]. However, despite the attractiveness of stainless steel devices, it is well-known that they are prone to localized corrosion in the human body due to chloride-induced pitting attack [7,8,9].

Additional concerns are related to parts subjected to relative movement arise, as they potentially damage the protective passive film of stainless steels [10]. The interaction of mechanical wear and chemical/electrochemical degradation is called tribocorrosion [11]. Such synergy frequently leads to more severe damage than the isolated effects [12]. In the biomedical field, wear debris generated during tribocorrosion, and metallic ions released due to dissolution of metallic surfaces, may cause implant failure due to tissue inflammation [13]. Stainless steel devices exhibit susceptibility to tribocorrosion in biomedical applications [14]. Degradation of the passive film corrosion protection ability is directly related to the deleterious effects of tribocorrosion. In this respect, breakdown and repassivation characteristics of the passive film depend on environmental aspects such as electrolyte composition and electrochemical potential [15,16]. Alkan and Gök [17] studied the effect of the applied potential on the tribocorrosion behavior of the AISI 316 L stainless steel in seawater. Depending on the electrochemical potential, deterioration of the metallic surface was intensified. The largest wear volumes were observed for anodic potentials. However, a detailed correlation between the composition of the passive film and the tribocorrosion behavior was not given. Zhang et al. [18] studied the effect of the electrochemical potential on the tribocorrosion behavior of the UNS S31254 super austenitic stainless steel in seawater. Corrosion-assisted wear dominated the tribocorrosion behavior at the more anodic potentials, due to more aggressive dissolution and pitting formation. The correlation between passive film composition and the results obtained in the tribocorrosion tests was not explored. Eghlimi et al. [19] investigated the tribocorrosion behavior of overlay welded super duplex stainless steel in chloride solution under open circuit and potentiostatic polarization conditions. Anodic potentials were found to accelerate material loss. Detailed assessment of the correlation between passive film composition and the tribocorrosion behavior was not provided. Tasdemir et al. [20] developed polyurethane and polyvinylpyrrolidone coatings for corrosion protection of 316 L stainless steel in sodium chloride solution. The tribocorrosion behavior was assessed by reciprocating wear tests. Information on the passive film stability of the uncoated substrate and its correlation with the tribocorrosion behavior was not given. Uzun [21] employed plasma nitriding to modify the surface of 316 L stainless steel samples and studied its tribocorrosion behavior in a simulated body fluid. The untreated substrate was subjected to more intense material loss during tribocorrosion tests. The passive film composition was not assessed, nor was its effect on the tribocorrosion behavior of the uncoated 316 L samples.

In spite of the highly recognized central role played by the passive film stability on the tribocorrosion behavior of stainless steels, detailed examination of passive film composition and its correlation with the corrosion-wear synergism of surgical austenitic stainless steel is not frequently reported in the literature. In the light of this scenario, the aim of the present work was to investigate the effect of the electrochemical potential on the tribocorrosion behavior of ASTM F139 stainless steel in phosphate-buffered saline solution (PBS), exploring the corrosion between the passive film composition and the observed wear/friction properties of the metallic surface. Tribocorrosion tests were conducted in a ball-on-disk tribometer. The passive film composition was analyzed using X-ray photoelectron spectroscopy (XPS).

## 2. Materials and Methods

### 2.1. Material and Sample Preparation

The ASTM F139 stainless steel plate (2000 mm × 1000 mm × 2 mm) (chemical composition in wt.%: Cr-17.93; Ni-14.42; Mo-2.88; Mn-1.79; Si-0.32; Cu-0.22; N-0.082; P-0.02; S-0.001; Fe-Bal) was purchased from Acnis (Sorocaba, Brazil). Disk-shaped specimens were milled from the original plate (Romi, S30) with a diameter of 90 mm. Surface finishing was accomplished by mechanical grinding with silicon carbide waterproof papers up to grit 1500. Surface roughness was measured with confocal laser scanning microscopy (Olympus, LEXT OLS4100, Tokyo, Japan), obtaining an average roughness (Ra) of 0.07 + 0.01 µm.

### 2.2. Potentiodynamic Polarization

Preliminary potentiodynamic polarization tests were initially conducted to examine the passive range of the ASTM F139 specimens, thus allowing definition of the applied potentials employed in the tribocorrosion tests. Hence, rectangular specimens with a 1 cm^2^ flat surface were connected to a copper wire using a conductive silver colloidal paste. Next, this set was embedded in cold curing epoxy resin. After complete curing, the specimens were ground up to grit 1500 using silicon carbide waterproof papers. Next, they were washed with distilled water and subjected to the electrochemical test. The tests were conducted using an Ivium n-Stat potentiotstat/galvanostat in a conventional three-electrode cell set-up. The ASTM F139 specimens were the working electrodes, a platinum wire (diameter of 0.5 mm) was used as the auxiliary electrode and Ag/AgCl (KCl, 3.0 M) was used as reference. Initially, the open circuit potential (OCP) was monitored for one hour. Next, potentiodynamic polarization curves were acquired by sweeping the potential from −0.3 V versus the OCP up to +1.0 V Ag/AgCl at a scan rate of 1 mV.s^−1^. The tests were conducted in phosphate buffered saline solution (PBS, 0.355 g.L^−1^ NaH_2_PO_4_·H_2_O, 8.2 g.L^−1^ NaCl, and 0.105 g.L^−1^ Na_2_HPO_4_, pH = 7.4; conductivity 17 mS.cm^−1^). The tests were conducted in triplicate.

### 2.3. Tribocorrosion Tests

**Figure 1:**
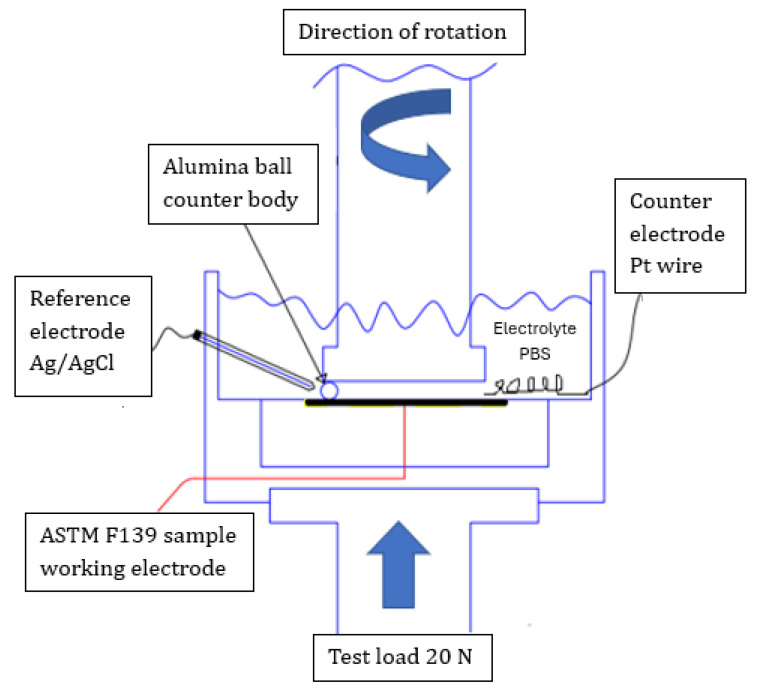
Schematic representation of the experimental set-up of the tribocorrosion tests for the ASTM F139 stainless steel samples in PBS solution.

Tribocorrosion tests were conducted in PBS, using a ball-on-disk tribometer (Ducom Instruments, Micro PoD, Bengaluru, India), connected with a three-electrode cell with the ASTM F-139 disk as the working electrode, a platinum wire as the auxiliary electrode, and Ag/AgCl (KCl, 3.0 M) as the reference electrode. The experimental set-up is schematically shown in Figure 1. Alumina balls (6 mm diameter) were used as counter-bodies. The rotating speed was 60 rpm and the normal load was 20 N. The wear track radius was 40 mm. The effect of the applied potential was assessed. Four different potentials were applied using an Ivium n-Stat potentiostat/galvanostat: (i) −100 mV versus the OCP; (ii) open circuit potential; (iii) +200 mV_Ag/AgCl_; (iv) +700 mV_Ag/AgCl_. Initially, the OCP (for the OCP condition) or the current density (for the cathodic or anodic potentials) was monitored for 600 s (without wear). Then, the tribometer was turned on and the corresponding potential (OCP condition), or current density (polarized conditions), were concomitantly registered for 3600 s. Next, in the final step, the tribometer was turned off and the potential (OCP condition) or current density (polarized conditions) were registered for 600 s to evaluate the recovery of the passive film. Dry sliding wear tests were also conducted to compare the results with the tribocorrosion tests, following the same experimental parameters (normal load of 20 N, wear track radius of 40 mm, 6 mm alumina balls as counter-bodies, and test duration of 3600 s; rotating speed of 60 rpm).

### 2.4. Characterization

The worn surfaces were examined using scanning electron microscopy (JSM-610LA, JEOL) and confocal laser scanning microscopy (CLSM—Olympus, LEXT OLS4100). The wear volume was estimated from the CLSM micrographs based on the procedure described by Kossman et al. [22]. Briefly, using CLSM, the section area of the wear track was measured at eight equally spaced regions distributed along its whole perimeter, taking the profiles perpendicular to the wear track. The average area was multiplied by the perimeter of the wear track circumference, thus obtaining the wear volume.

The chemical composition of the passive film, before and after the wear tests, was assessed using X-ray photoelectron spectroscopy (XPS). A ThermoFisher Scientific (K-alpha+) spectrometer, equipped with Al-kα monochromatic X-ray source, was used for the analysis. The spot size was 400 µm. The pressure in the analysis chamber was 10^−7^ Pa. The pass energies were 200 eV and 50 eV for the survey and high resolution spectra, respectively. The Smart algorithm was used for background subtraction. Peak fitting was accomplished in the Avantage© software (version 5.976) with a mixture of Lorentzian–Gaussian functions (70/30). Depth profile experiments were conducted to evaluate the distribution of different elements throughout the thickness of the passive film.

## 3. Results and Discussion

### 3.1. Static Polarization

**Figure 2:**
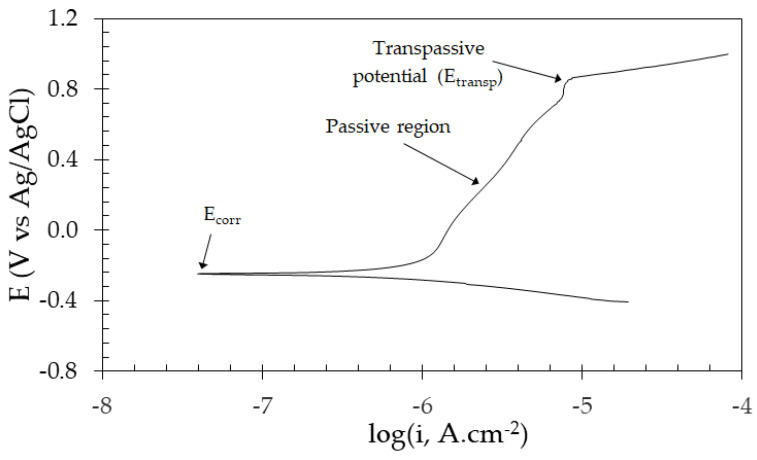
Potentiodynamic polarization curve of the ASTM F139 stainless steel after 1 h of immersion in PBS solution.

Initially, static polarization tests were conducted to allow the selection of the electrochemical potentials that would be employed during the tribocorrosion tests (cathodic, open circuit, and anodic conditions). Figure 2 displays one representative potentiodynamic polarization curve obtained for the ASTM F139 stainless steel after 1 h of immersion in PBS solution. The material exhibited a typical passive behavior. The corrosion potential (E_corr_) was −220 mV_Ag/AgCl_. In the anodic branch of the polarization curve, the transpassive potential (E_transp_) was approximately +900 mV_Ag/AgCl_. Hence, the passive range (ΔE = E_transp_ − E_corr_) is 1120 mV. From this result, the electrochemical potentials employed in the tribocorrosion tests were defined as follows: (i) −100 mV versus the open circuit potential (cathodic potential); (ii) open circuit potential (OCP); (iii) +200 mV (anodic potential, in the middle of the passive range); (iv) +700 mV (anodic potential, near the transpassive potential).

### 3.2. Tribocorrosion Tests

#### 3.2.1. Sliding Wear under Open Circuit Condition

**Figure 3:**
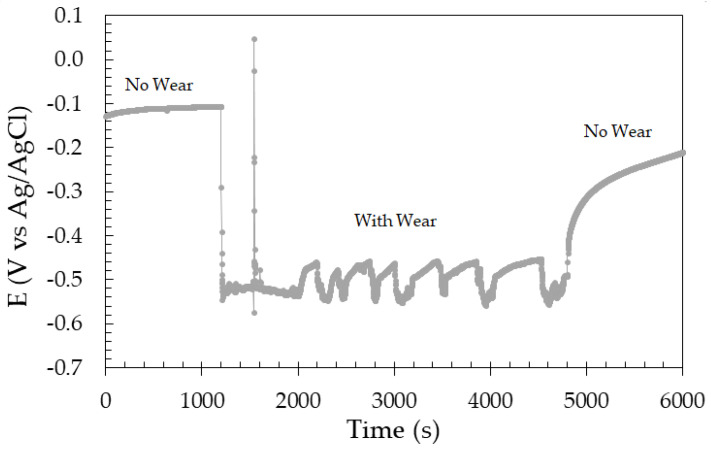
Evolution of the open circuit potential of the ASTM F139 stainless steel before, during, and after sliding wear in PBS solution.

Initially, the open circuit potential (OCP) of the ASTM F139 stainless steel was monitored before, during, and after sliding wear in PBS solution. The results are displayed in Figure 3.

Before initiating the sliding step of the test, the OCP was approximately −120 mV versus Ag/AgCl. Right after the beginning of the sliding test, the OCP exhibited an immediate drop to a more cathodic potential of −530 mV versus Ag/AgCl. This behavior is typically observed for passive metals under sliding wear tribocorrosion tests, due the removal of the surface oxide film [23,24]. Thus, a galvanic effect takes place between the undamaged passive film in the surroundings of the wear track, and the damaged passive film inside the wear track, intensifying the corrosion process [25]. The OCP fluctuations during sliding wear are associated with the continuous destruction and recovery of the passive film, but without the ability to completely regenerate due to the mechanical removal [26,27]. As the sliding wear step of the test ends, the OCP is shifted in the anodic direction, indicating recovery of the passive film within the wear track. However, the OCP value was lower than that of the undamaged passive film before the sliding wear.

#### 3.2.2. Effect of Applied Potential on the Tribocorrosion Behavior of the ASTM F139 Stainless Steel

**Figure 4:**
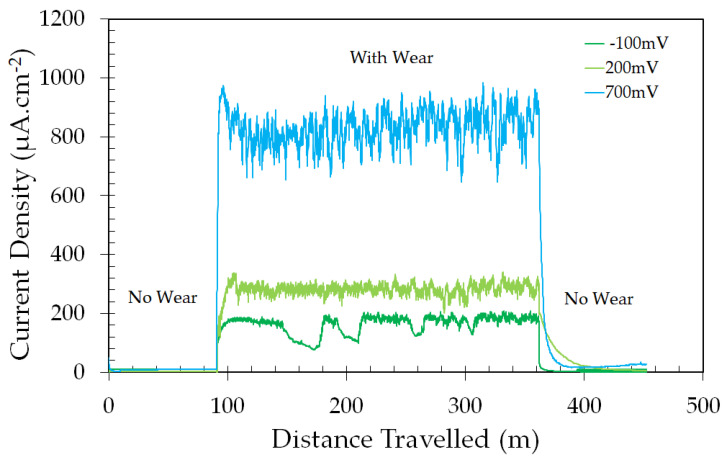
Evolution of the current density during potentiostatic tribocorrosion tests of the ASTM F139 stainless steel in PBS solution.

Figure 4 shows the evolution of the current density during potentiostatic tribocorrosion tests of the ASTM F139 stainless steel in PBS solution. The current densities were stable before the sliding wear step, as has also been reported by other authors for different passive metals [28,29]. The sample subjected to cathodic potential (−100 mV versus OCP) exhibited the lowest current densities, which were progressively increased as the applied potential became more anodic. A sharp increase of the current density occurred when the sliding wear initiated, which was dependent on the applied potential. The highest values were observed for the potential of +700 mVAg/AgCl, the most anodic potential employed in the current work. Hence, anodic polarization led to intense dissolution of the ASTM F139 stainless steel specimen during tribocorrosion tests. As the sliding wear step ended, the current densities immediately dropped due to passive film regeneration. The cathodic potential displayed the lowest current density at this final stage. For the anodic applied potentials, the current densities were not as stable as the cathodic condition, gradually increasing up to the end of the test.

#### 3.2.3. Effect of Applied Potential on the Frictional Behavior

**Figure 5:**
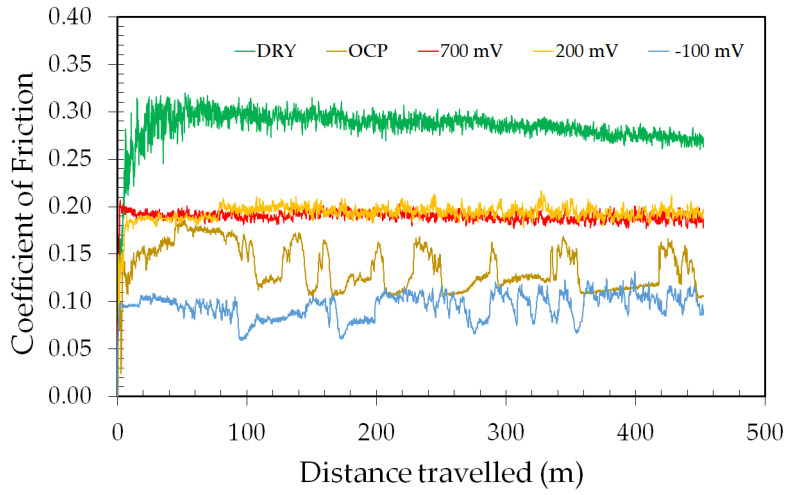
Evolution of COF with the sliding distance for ASTM F139 stainless steel samples at different applied potentials.

The evolution of the coefficient of friction (COF) with the sliding distance for ASTM F139 stainless steel samples subjected to different electrochemical potentials is displayed in Figure 5.

The dry sliding condition exhibited the highest COF values, reaching approximately 0.25 at the end of the test. The COF values were lower for the specimens subjected to the tribocorrosion tests, due to the well-known lubricating action of the electrolyte, as reported by several authors [30,31,32]. The COF value of the OCP condition is higher than the cathodic potential of −100 mV versus OCP. According to Alkan and Gök [17], the cathodic condition may decrease the COF value with respect to the OCP condition, as the metallic surface is only subjected to mechanical wear, and the simultaneous action of corrosion processes does not take place. Conversely, as the potential becomes more anodic, the COF increased due to the corrosion action at the wear track, forming corrosion products concomitantly to wear debris, thus raising the frictional response at the contact between the counter-body and the sample surface, as observed by Zhang et al. [18] for the S31254 steel in seawater.

#### 3.2.4. Wear Volume

**Figure 6:**
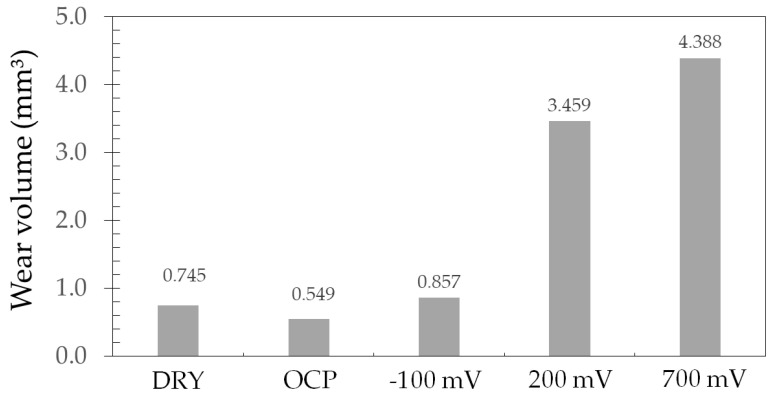
Wear volume of the ASTM F139 stainless steel samples after tribocorrosion tests in PBS solution.

The wear volume of the ASTM F139 samples after tribocorrosion tests in PBS solution was estimated based on the work by Kossman et al. [22]. The results are displayed in Figure 6. The result obtained for the dry sliding test is included for comparison.

According to the results shown in Figure 6, there is a trend of increasing the wear volume with the applied potential. The anodic potentials exhibited the highest wear volumes. According to the literature [33], this behavior can be explained by two distinct factors. Firstly, the removal of the passive film during sliding wear enhances dissolution of the metallic surface. Secondly, the plastic deformation in the wear track during the tribocorrosion tests increases the density of defects, such as cracks and dislocations, making this region active which, in turn, accelerates corrosion. Additionally, wear can be envisaged as a low-cycle fatigue process during tribocorrosion. Hence, the breakdown and removal of the passive film reduces the real contact area during sequential cycles of the wear process, thus increasing contact stresses and, ultimately, the wear volume. Corrosion greatly accelerated the mass loss with respect to the pure wear effect of the dry sliding condition, as also observed by other authors [18].

### 3.3. Scanning Electron Microscopy

**Figure 7:**
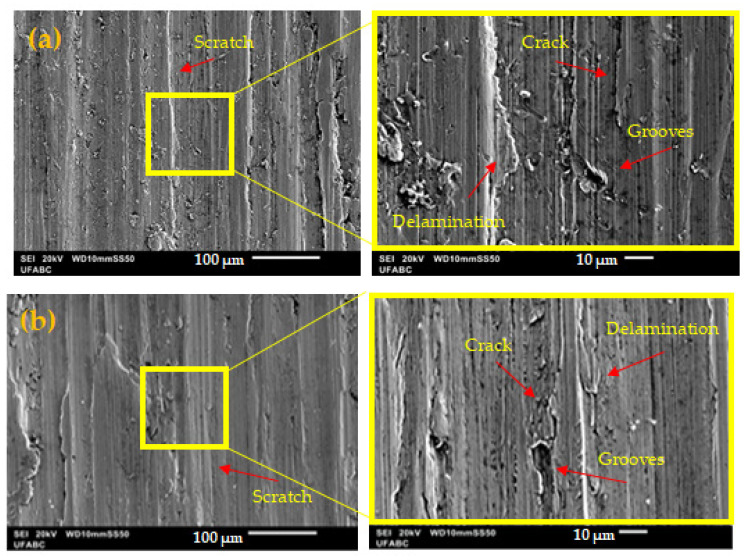
SEM micrographs of the wear tracks of the ASTM F139 stainless steel specimens after tribocorrosion tests in PBS solution: (**a**) Dry sliding; (**b**) OCP.

**Figure 8:**
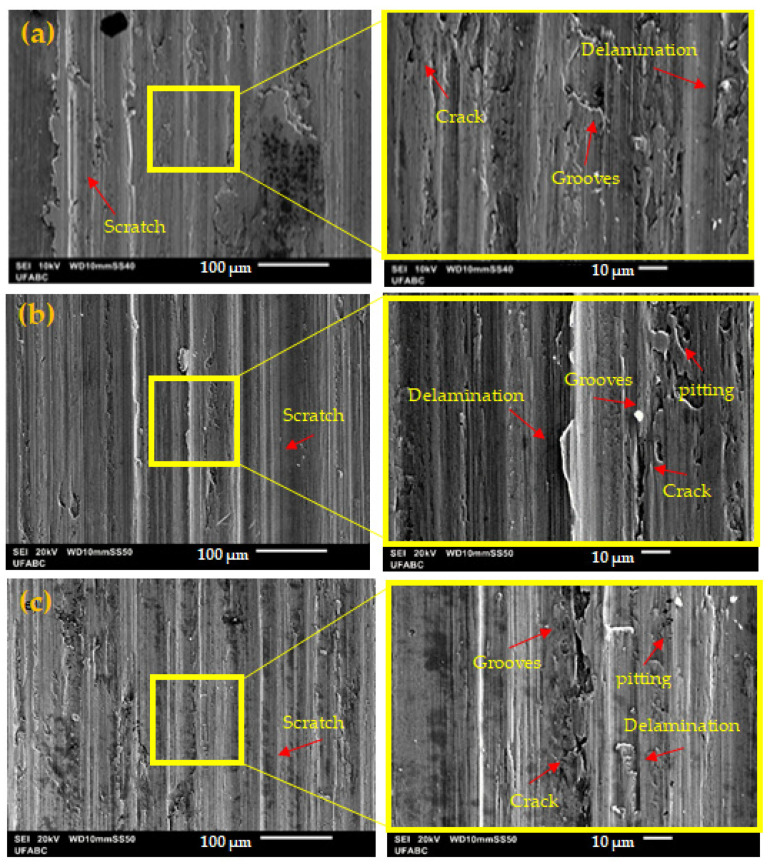
SEM micrographs of the wear tracks of the ASTM F139 stainless steel samples after tribocorrosion tests in PBS solution: (**a**) −100 mV vs. OCP; (**b**) +200 mV vs. Ag/AgCl; (**c**) +700 mV vs. Ag/AgCl.

SEM micrographs of the wear tracks of the ASTM F139 stainless steel specimens subjected to tribocorrosion tests in PBS solution at different applied potentials are shown in Figure 7 and Figure 8. The wear tracks of all samples presented signs of plastic deformation, indicating the prevailing action of abrasive wear throughout the tests, due to the contact of the ductile metallic sample with the hard ceramic alumina ball [18,34].

In the dry sliding condition (Figure 7a), the wear track morphology exhibits evident signs of mechanical damage, such as scratches, grooves, cracks, and delaminated regions. For the OCP sample (Figure 7b), the wear track displayed a smoother morphology, with fewer cracks and wear debris than the dry sliding specimen. The lubricating action of the electrolyte is probably associated with the less defective wear track morphology, in agreement with the reduced wear volume of the OCP specimen when compared to the other ones, as seen in Figure 6.

The micrograph of the wear track of the specimen subjected to the cathodic potential (−100 mV versus the OCP) is shown in Figure 8a. The worn surface is relatively smooth when compared to the dry sliding and OCP specimens (Figure 7a and Figure 7b, respectively), although some grooves were formed, as can be seen in the higher magnification image shown on the right. The cathodic potential inhibits the formation of corrosion products during the tribocorrosion test which, otherwise, could participate in the corrosion–wear interaction. The corrosion products may act as lubricating particles at the point of wear contact, making the worn surface smoother. As outlined by Zhu et al. [35], as cathodic potentials inhibit the formation of lubricating corrosion products, the wear volume is likely to increase with respect to the OCP condition. The same result was observed in the present work, as seen in Figure 6.

Anodic polarization greatly increased the wear volume of the ASTM F-139 samples, as highlighted in the previous section. The worn surfaces of the samples subjected to anodic polarization are shown in Figure 8b (+200 mV vs. Ag/AgCl) and Figure 8c (+700 mV vs. Ag/AgCl). At +200 mV vs. Ag/AgCl, some pits were found in the wear track, as well as grooves, cracks, and delaminated areas. When the potentiostatic potential was increased to +700 mV vs. AgCl, more profound grooves and large cracks were formed, and pits were also found. The degradation signs in the worn surface of the anodically polarized samples denote the more severe dissolution of the ASTF F-139 alloy when compared to the OCP and cathodic conditions (Figure 7b and Figure 8a, respectively). Moreover, local corrosion spots associated with pitting corrosion are the source of corrosion products that may be easily removed during sliding movement, facilitating wear action at the metallic surface, thus increasing the wear volume, as reported in the literature for anodically polarized stainless steel samples [35]. This effect is seen in Figure 6, as revealed by the progressively higher wear volume when the anodic potential was increased from +200 mV to +700 mV vs. Ag/AgCl. The wear track morphologies shown in Figure 8b,c agree with this finding.

### 3.4. X-ray Photoelectron Spectroscopy (XPS)

**Figure 9:**
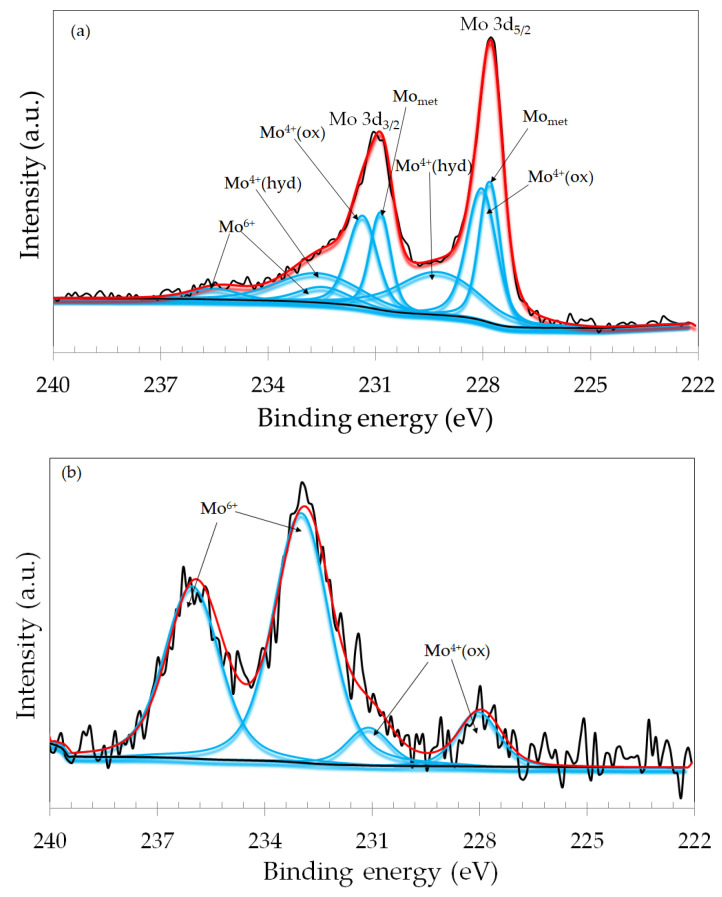

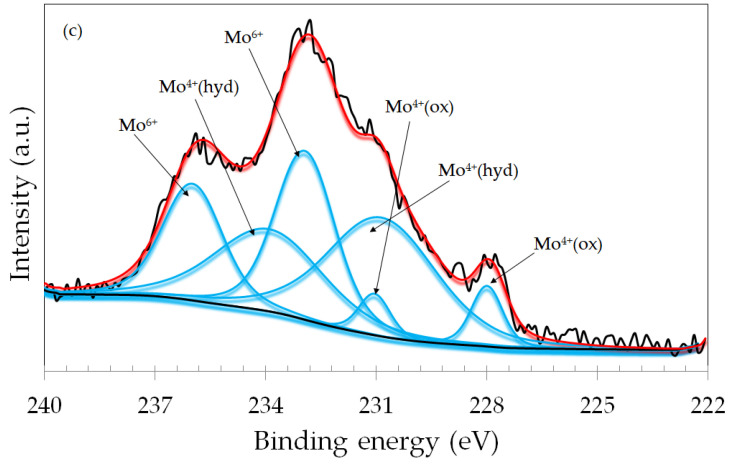
XPS narrow scan spectra for the Mo 3d photoelectron line of the ASTM F-139 samples (inside the wear track): (**a**) dry sliding; (**b**) +200 mV vs. Ag/AgCl; (**c**) +700 mV vs. Ag/AgCl.

XPS analysis of the wear tracks was carried out to assess the surface chemical states of the ASTM F-139 stainless steel samples subjected to the tribocorrosion tests at different applied potentials. The surface chemical states of the naturally formed passive film were also assessed for comparison purposes. The narrow scan spectra of the Mo 3d photoelectron lines of the dry sliding, +200 mV vs. Ag/AgCl and +700 mV vs. Ag/AgCl specimens, are shown in Figure 9. For the dry sliding specimen, the Mo 3d spectrum was fitted with four spin–orbit doublets that were assigned to the Mo 3d_5/2_ and Mo 3d_3/2_ branches of the following species: metallic Mo (Mo_met_), Mo^4+^ (oxide), Mo^4+^ (hydroxide), and Mo^6+^, in line with the literature [36,37]. The spectra obtained for the naturally formed passive film, OCP and −100 mV vs. OCP samples, exhibited the same components as those of the dry sliding one, and are displayed in the Supplementary Materials (Figure S1). Different spectra were obtained for the samples subjected to anodic potentials during the tribocorrosion tests. As shown in Figure 9b, the Mo 3d spectrum of the +200 mV vs. Ag/AgCl specimen was fitted with only two spin–orbit doubles which were assigned to Mo^4+^ (oxide) and Mo^6+^. The signals of metallic Mo and Mo^4+^ (hydroxide) were not detected. Moreover, the Mo^6+^ signal was much stronger than that of Mo^4+^, revealing the increasing oxidation character of the surface when compared to the dry sliding specimen (Figure 9a). For the specimen subjected to +700 mV vs. Ag/AgCl the Mo 3d spectrum was fitted with three spin–orbit doublets, as shown in Figure 9c, which were assigned to Mo^4+^ (oxide), Mo^4+^ (hydroxide), and Mo^6+^, with predominance in the Mo^4+^ species. The metallic component was also not present, as for the +200 mV vs. Ag/AgCl specimen.

**Figure 10:**
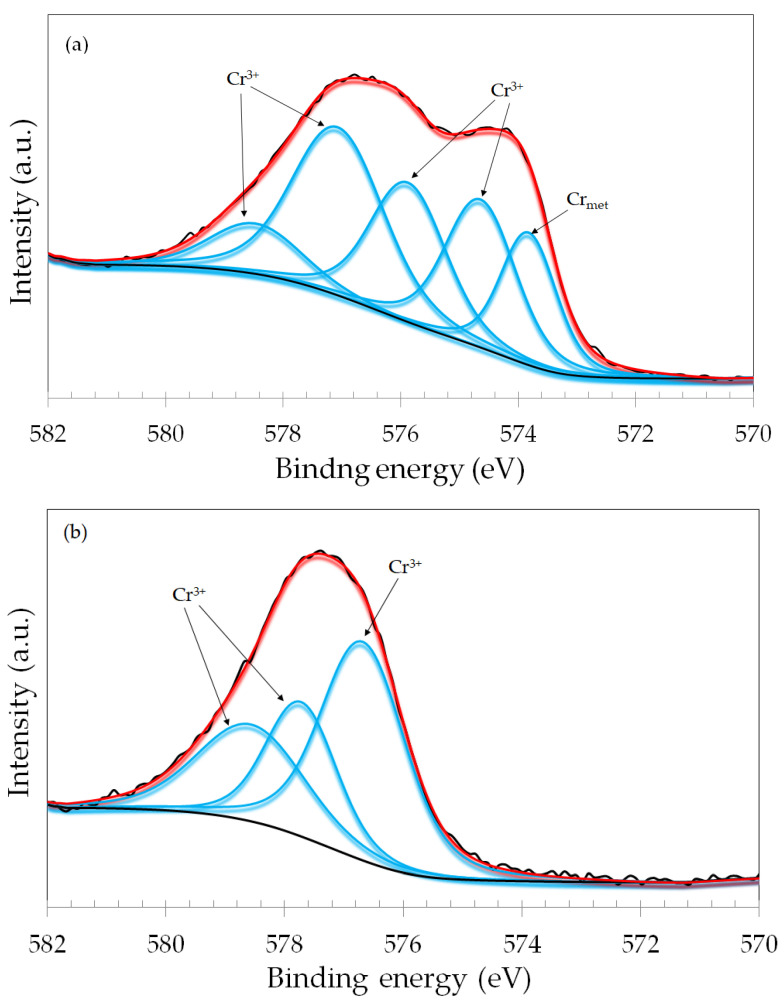

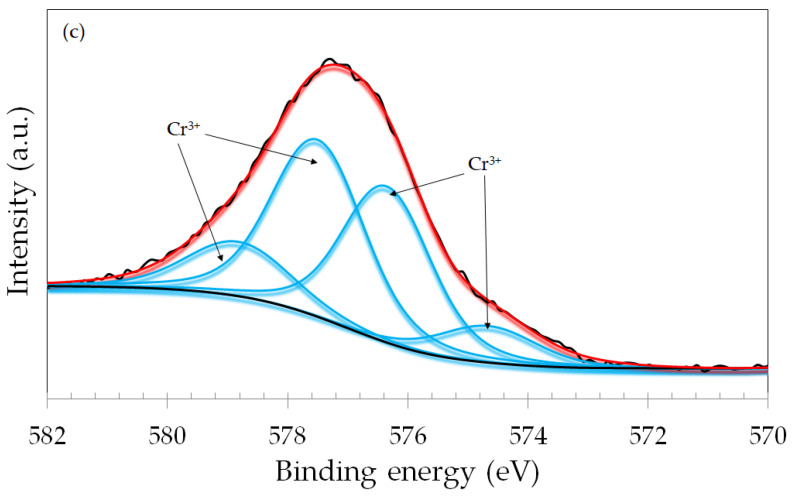
XPS narrow scan spectra for the Cr 2p_3/2_ photoelectron line of the ASTM F-139 samples (inside the wear track): (**a**) dry sliding; (**b**) +200 mV vs. Ag/AgCl sample; (**c**) +700 mV vs. Ag/AgCl sample.

The Cr 2p_3/2_ photoelectron lines of the dry sliding, +200 mV vs. Ag/AgCl and +700 mV vs. Ag/AgCl specimens, are shown in Figure 10. For the dry sliding specimen (Figure 10a), the spectrum was deconvoluted considering a mixture of metallic (Cr_met_) and Cr^3+^ (Cr_2_O_3_ and Cr(OH)_3_) species. Similar spectra were obtained for the naturally formed passive film, OCP and −100 mV vs. OCP specimens. They are exhibited in Figure S2 (Supplementary Materials). The Cr 2p spectrum displayed a typical multiplet splitting for Cr_2_O_3_, as reported by other authors [38,39]. The spectra of the specimens subjected to anodic potentials are different. As seen in Figure 10b for the +200 mV vs. Ag/AgCl sample the signal of metallic Cr was not observed. The spectrum was fitted with multiple peaks assigned only to Cr^3+^ species. Similarly, for the +700 mV vs. Ag/AgCl sample (Figure 10c), only Cr^3+^ species were present in the Cr 2p_3/2_ spectrum, and metallic Cr was not detected.

**Figure 11:**
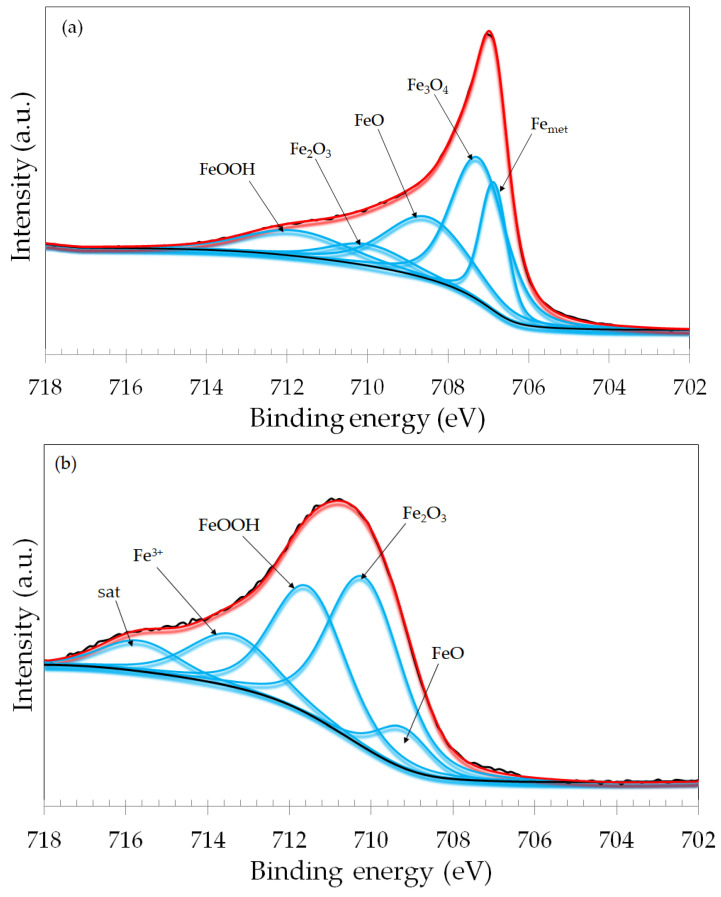

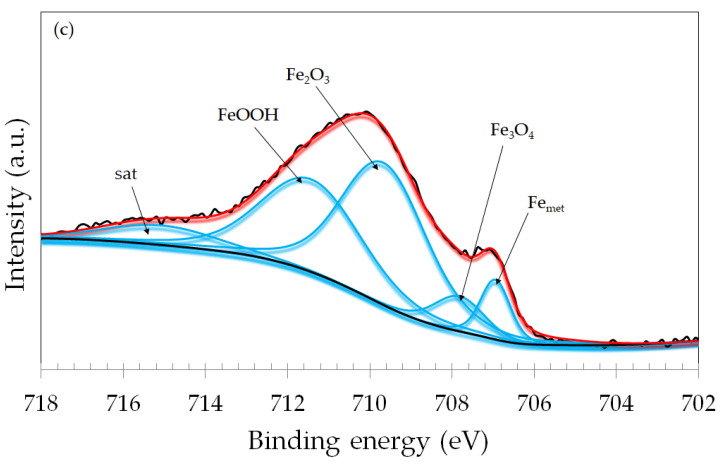
XPS narrow scan spectra for the Fe 2p_3/2_ photoelectron line of the ASTM F-139 samples (inside the wear track): (**a**) dry sliding; (**b**) +200 mV vs. Ag/AgCl; (**c**) +700 mV vs. Ag/AgCl.

The narrow scan spectra of Fe 2p_3/2_ are shown in Figure 11. The spectrum of the dry sliding specimen (Figure 11a) was fitted with five peaks that were assigned to metallic Fe (Fe_met_), and a mixture of Fe^2+^ (FeO) and Fe^3+^ (Fe_3_O_4_, Fe_2_O_3_ and FeOOH) species. The binding energies are in good agreement with the literature [40,41]. The spectra obtained for the naturally formed passive film, OCP and -100 mV vs. OCP specimens, were very similar, and are displayed in the Supplementary Materials (Figure S3). The samples subjected to anodic potentials, in turn, exhibited different Fe 2p_3/2_ spectra. The spectrum of the +200 mV vs. Ag/AgCl specimens (Figure 11b) was fitted with a mixture of Fe^2+^ and Fe^3+^ species. The signal of metallic Fe was not observed. For the +700 mV vs. Ag/AgCl specimen, a weak signal of metallic Fe was detected at the lowest binding energy, but the major species are assigned to Fe^3+^ compounds. The results indicate that the anodic potentials increased the relative fraction of Fe^3+^ species in the surface oxide film, leading it to a higher oxidation condition.

**Figure 12:**
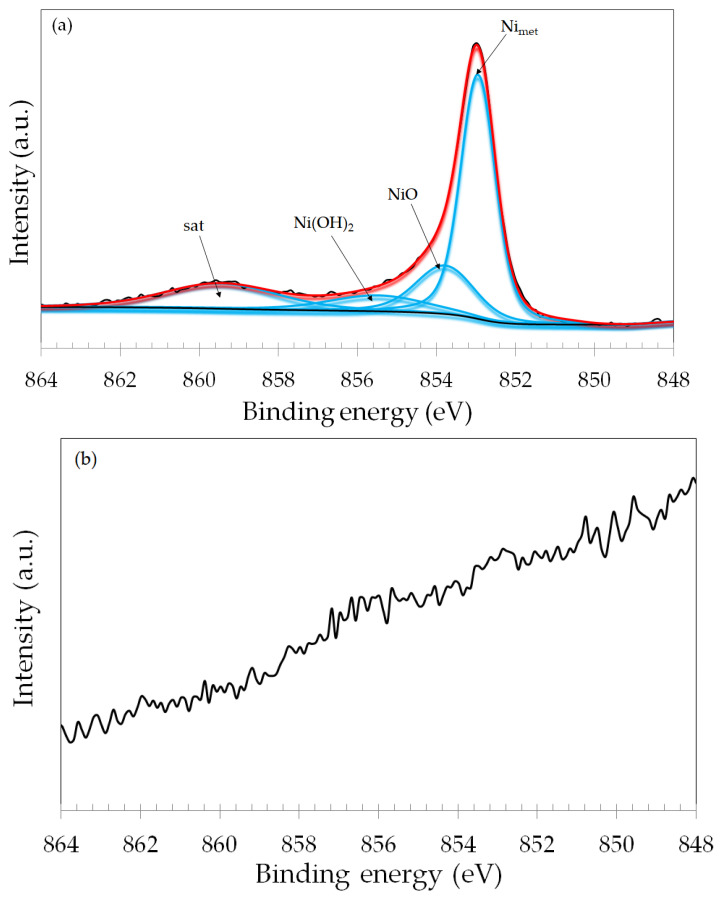

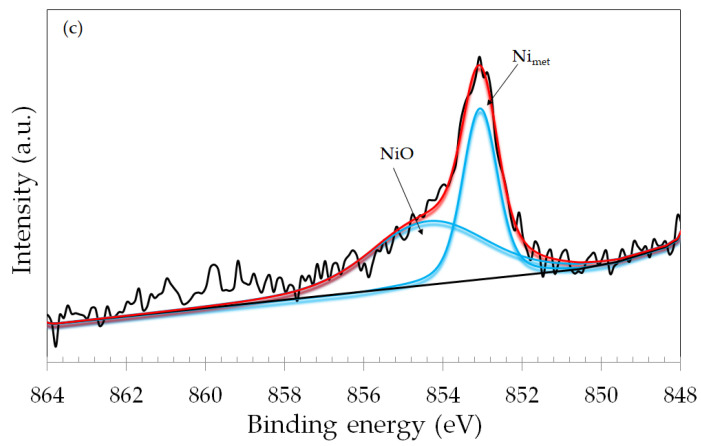
XPS narrow scan spectra for the Ni 2p_3/2_ photoelectron line of the ASTM F-139 samples (inside the wear track): (**a**) dry sliding; (**b**) +200 mV vs. Ag/AgCl; (**c**) +700 mV vs. Ag/AgCl.

Additional information on the composition of the oxide film on the wear tracks was obtained by assessing the Ni 2p_3/2_ narrow scan spectra (Figure 12). For the dry sliding specimen (Figure 12a), the spectrum was fitted with three main components and a high-energy satellite. The main component was assigned to metallic Ni (Ni_met_). NiO and Ni(OH)_2_ were also observed, with lower intensities. The binding energies are in good agreement with the literature [42,43].

The predominance of metallic Ni over its oxidized species is often reported in the literature, due to the relatively low oxidation susceptibility of Ni compared to Cr and Fe [44]. The Ni 2p_3/2_ spectra of the naturally formed passive film, OCP and −100 mV vs. OCP specimens, displayed similar features, and are shown in the Supplementary Materials (Figure S4). The spectrum of the +200 mV vs. Ag/AgCl specimen was completely different. Indeed, the signal of Ni 2p_3/2_ was negligible for this sample, as seen in Figure 12b. Hence, it is possible to infer that Ni species are not present in the oxide film formed in this wear track. For the +700 mV vs. Ag/AgCl (Figure 12c), although the Ni signal is lower than the one observed in the spectrum of the dry sliding specimen (Figure 12a), it is much more intense than for the +200 mV vs. Ag/AgCl wear track. Thus, it was possible to fit the spectrum with two peaks that were assigned to metallic Ni and NiO.

**Figure 13:**
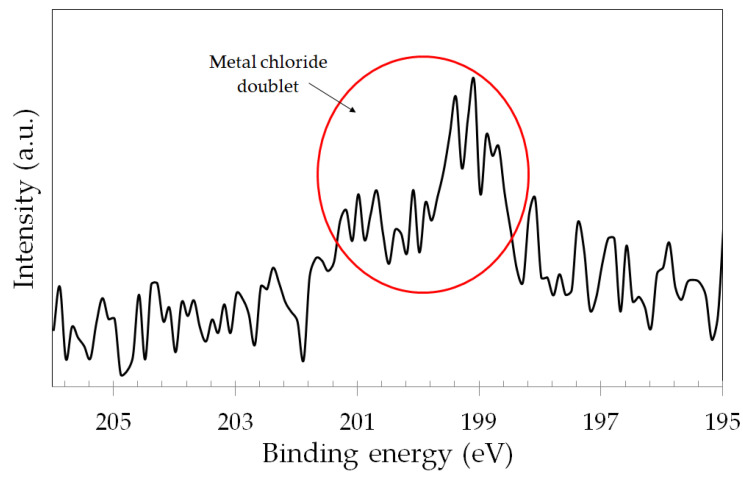
XPS narrow scan spectra for the Cl 2p photoelectron line of the ASTM F-139 stainless steel sample (inside the wear track) in the OCP condition.

As the samples were in contact with the PBS solution during the tribocorrosion tests, chloride species were likely to be incorporated into the surface oxide film. This possibility was checked by assessing the Cl 2p narrow scan spectra for the samples subjected to the simultaneous action of wear and corrosion (OCP, +200 mV vs. Ag/AgCl, +700 mV vs. Ag/AgCl and −100 mV vs. OCP). Figure 13 shows a representative Cl 2p spectrum for the OCP specimen. The spectra of the other samples displayed similar characteristics and are available in the Supplementary Materials (Figure S5). The signal of the Cl 2p photoelectron line is relatively weak for all samples. Nonetheless, a typical metal chloride peak is clearly seen, as indicated in Figure 13, in accordance with other published results [45,46].

**Figure 14:**
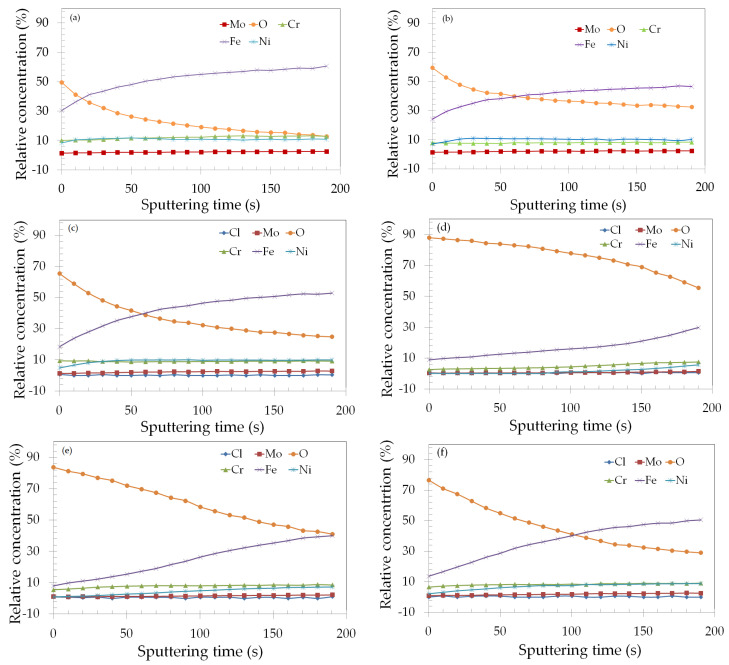
XPS Depth profiles of the main elements in the passive films of the ASTM F-139 stainless steel samples after dry sliding and tribocorrosion tests: (**a**) naturally formed passive film; (**b**) wear track—dry sliding sample; (**c**) OCP; (**d**) +200 mV vs. Ag/AgCl; (**e**) +700 mV vs. Ag/AgCl; (**f**) −100 mV vs. OCP.

From the XPS narrow scan spectra of the main metallic elements in the wear tracks (Figure 9, Figure 10, Figure 11 and Figure 12), the different composition of the samples subjected to anodic potentials was unequivocally shown. The signals of the metallic components were absent or relatively weak compared to the samples tested under OCP or the cathodic potential. To gain further understanding of the reason for such difference, XPS depth profile experiments were also conducted. The results are displayed in Figure 14 for all samples, showing the variation of the relative concentrations of Mo, Cr, Fe, Ni, and O with the sputtering time. For the samples subjected to the tribocorrosion tests, the Cl concentration was also assessed. The composition depth profile of the naturally formed passive film (Figure 14a) reveals a relative concentration of O close to 50%. After the first three sputtering cycles, the iron concentration surpasses that of oxygen, as the passive film is gradually removed. Cr and Ni concentrations are close to each other, while Mo is present at lower concentrations, which do not vary significantly, up to the end of the experiment. The main difference of this sample to the depth profile of the dry sliding wear track (Figure 14b) is that the oxygen concentration was higher at the beginning of the experiment (approximately 60%), and the iron concentration only surpassed it after six sputtering cycles, indicating that the oxide film was thicker. The depth profile of the OCP specimen (Figure 14c) exhibits similar variations of the oxygen and iron concentrations with the sputtering time, but the oxygen concentration was slightly higher at the beginning of the experiment. Additionally, a very low Cl concentration was detected, but it practically disappeared after the first sputtering cycle, indicating that metal chlorides were adsorbed at the very first top surface of the oxide film. The same variation of Cl was also observed for the other samples subjected to the tribocorrosion tests (Figure 14d–f).

The cathodically polarized specimen (Figure 14f) exhibited a similar trend for the oxygen and iron concentrations compared to the OCP (Figure 14c), but with a higher oxygen concentration at the beginning of the experiment. Moreover, the iron concentration only surpassed oxygen after ten sputtering cycles (approximately 100 s), indicating that the oxide film was thicker for this sample.

The main differences, though, were observed for the anodically polarized samples. As seen in Figure 14d (+200 mV vs. Ag/AgCl), the oxygen concentration was greatly increased with respect to the OCP and the cathodically polarized samples. The iron concentration did not surpass oxygen up to the end of the experiment and was approximately 47% lower after the last sputtering cycle. Such variation indicates that the +200 mV vs. Ag/AgCl specimen was greatly oxidized during the tribocorrosion test. This would explain the absence of the signal of metallic Mo, Cr, Fe, and Ni in the XPS narrow scan spectra of this specimen (Figure 9b, Figure 10b, Figure 11b and Figure 12b). For the +700 mV vs. Ag/AgCl specimen (Figure 14e), the iron concentration does not surpass that of oxygen up to the end of the experiment, but it is not as low as in Figure 14d. This result indicates that the oxide film in the wear track was thinner for the specimen anodically polarized at +700 mV_Ag/AgCl_. This would explain why the weak signals of metallic iron and nickel were observed for this specimen and not for the specimen polarized at +200 mV_Ag/AgCl_.

The wear volumes (Figure 6) were greatly increased for the anodically polarized samples, indicating the relevant contribution of corrosion to the total removal of material, with respect to the pure mechanical wear (dry sliding sample). As observed by XPS depth profile experiments, highly oxidized surfaces were obtained for these samples. They also showed the predominance of the high oxidation states of the main metallic elements that constitute the surface oxide film.

## 4. Conclusions

In the present work, the tribocorrosion behavior of the ASTM F-139 surgical austenitic stainless steel was studied using ball-on-disc wear tests and electrochemical measurements taken in PBS solution. The effect of the applied potential on the wear volume was evaluated, as well as the composition of the surface oxide film. The following conclusions can be drawn from the obtained results:

- The applied potential had a remarkable effect on the tribocorrosion behavior of the ASTM F-139 stainless steel samples. In the OCP condition and the cathodic potential (−100 mV vs. OCP), the wear volume and coefficient of friction were reduced when compared to the dry sliding test. The anodic potentials (+200 mV and +700 mV vs. Ag/AgCl) greatly increased the wear volume. In this case, the coefficient of friction was higher than for the OCP and the cathodic potential, although it was also lower than in the dry sliding condition. The lubricant action of the PBS electrolyte during the tribocorrosion tests was responsible for the reduced coefficient of friction;
- The current densities measured during potentiostatic polarization were greatly increased for the anodic applied potentials. The wear volumes were correspondingly much higher, revealing the strong dissolution effect and the enhanced contribution of corrosion to the total wear volume;
- The morphology of the wear tracks was affected by the applied potential. For the anodic potentials, in addition to mechanical damage (cracks, grooves, scratches, and delaminated regions), corrosion pits were also observed;
- XPS analysis gave details about the composition of the oxide film on the wear tracks. Similar spectra were obtained for the naturally formed passive film, the films formed on the surface of specimens subjected to the dry sliding, and the OCP and cathodic conditions. The surface film was mainly composed of a mixture of Fe and Cr oxides, with minor amounts of Ni and Mo oxidized species. The signals of metallic Mo, Cr, Fe, and Ni were also detected. For the anodic potentials, in turn, the surface was more oxidized, especially for the +200 mV vs. Ag/AgCl specimen. Depth profiles were useful to confirm the strongly oxidized nature of the surface films formed on the specimens subjected to anodic potentials.

## Data Availability

The data presented in this study are available on request from the corresponding author.

## Supplementary Materials

The following supporting information can be downloaded at: https://www.mdpi.com/article/10.3390/ma17102295/s1, Figure S1: XPS narrow scan spectra in the Mo 3d region: (a) naturally formed passive film; (b) wear track of the OCP sample; (c) wear track of the −100 mV vs. OCP. Figure S2: XPS narrow scan spectra in the Cr 2p_3/2_ region: (a) naturally formed passive film; (b) wear track of the OCP sample; (c) wear track of the −100 mV vs. OCP. Figure S3: XPS narrow scan spectra in the Fe 2p_3/2_ region: (a) naturally formed passive film; (b) wear track of the OCP sample; (c) wear track of the -100 mV vs. OCP. Figure S4: XPS narrow scan spectra in the Ni 2p_3/2_ region: (a) naturally formed passive film; (b) wear track of the OCP sample; (c) wear track of the −100 mV vs. OCP. Figure S5: XPS narrow scan spectra in the Cl 2p region: (a) +200 mV vs. Ag/AgCl; (b) +700 mV vs. Ag/AgCl; (c) −100 mV vs. OCP.
