# Supplementary material for: Exploring the Interplay between Tribocorrosion and Surface Chemistry of the ASTM F139 Surgical Stainless Steel in Phosphate-Buffered Saline Solution
Source: Materials (Basel). 2024 May 13;17(10):2295. doi: 10.3390/ma17102295 (PMC11123090; doi:10.3390/ma17102295)
Supplement: Supplementary file 1 [file materials-17-02295-s001.zip › materials-2975803-supplementary.pdf]

Supplementary Materials

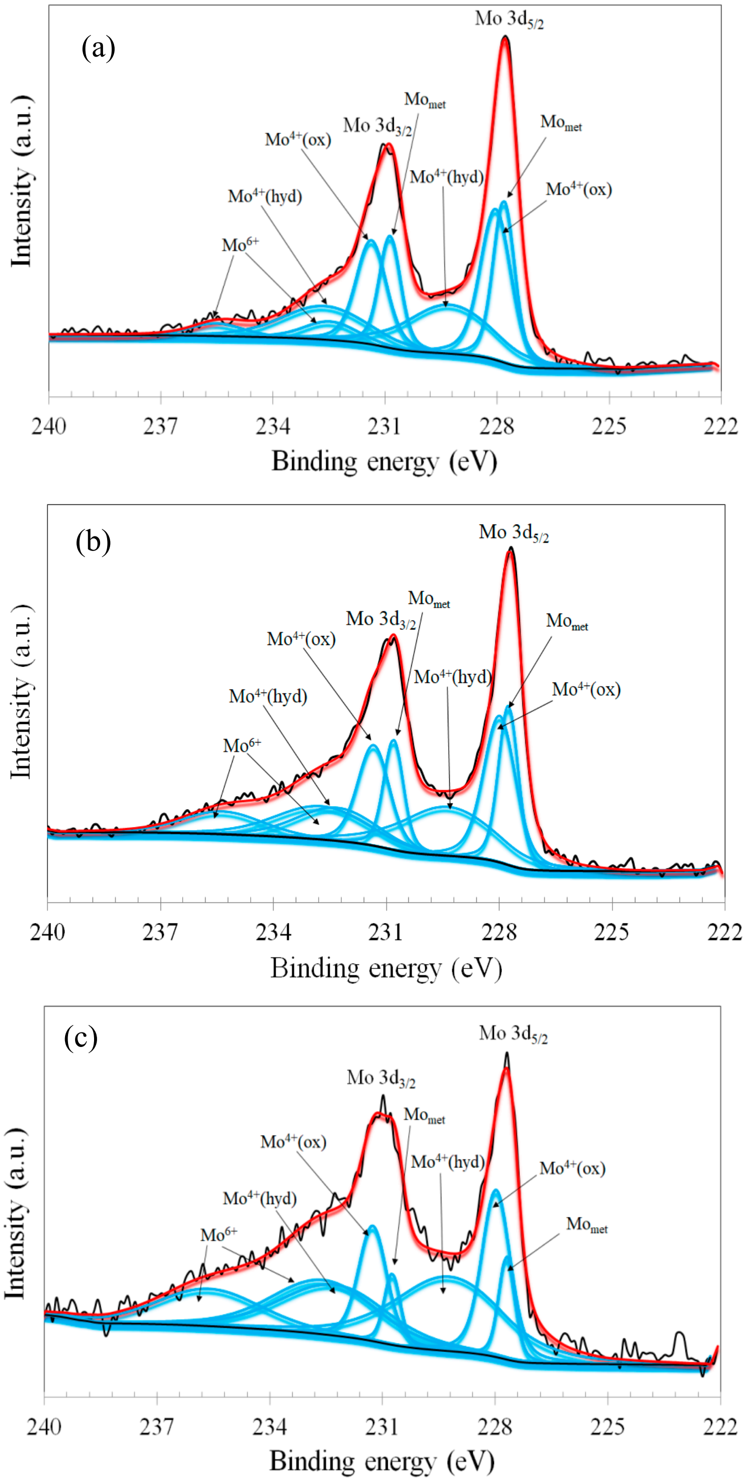

Figure S1. XPS narrow scan spectra in the Mo 3d region: a) naturally formed passive film; b) wear track of the OCP sample; c) wear track of the -100 mV vs. OCP.

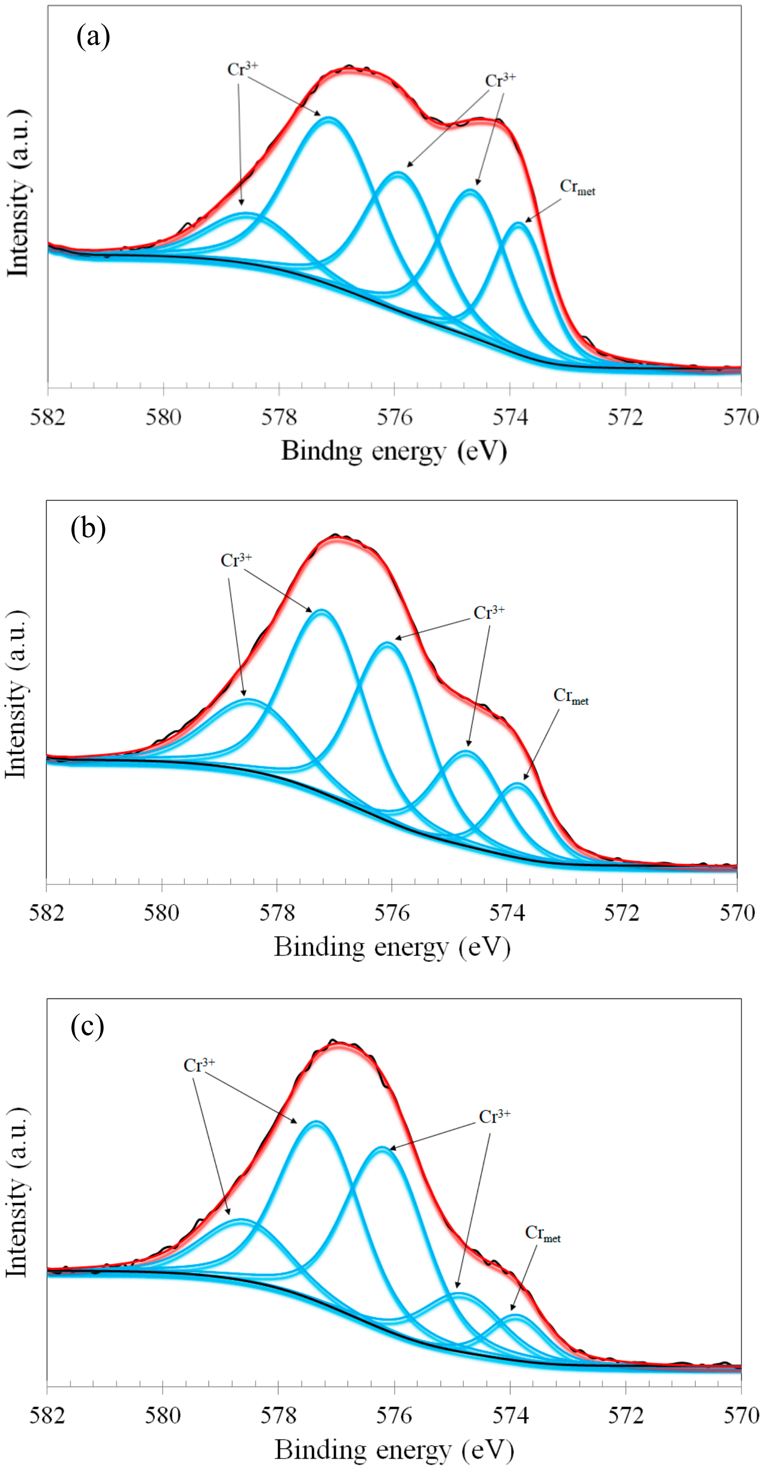

Figure S2. XPS narrow scan spectra in the Cr $2p_{3/2}$ region: a) naturally formed passive film; b) wear track of the OCP sample; c) wear track of the -100 mV vs. OCP.

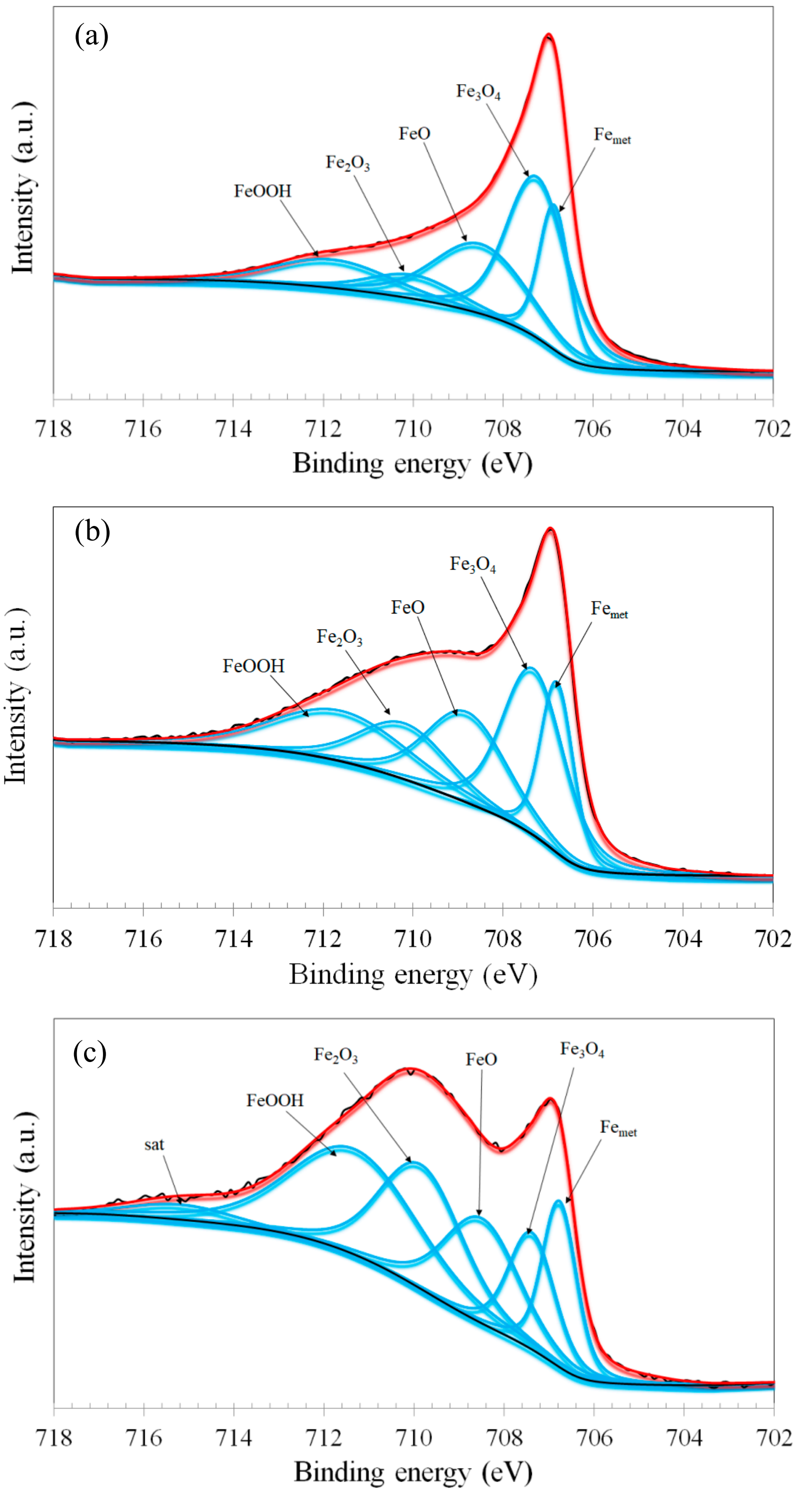

Figure S3. XPS narrow scan spectra in the Fe $2p_{3/2}$ region: a) naturally formed passive film; b) wear track of the OCP sample; c) wear track of the -100 mV vs. OCP.

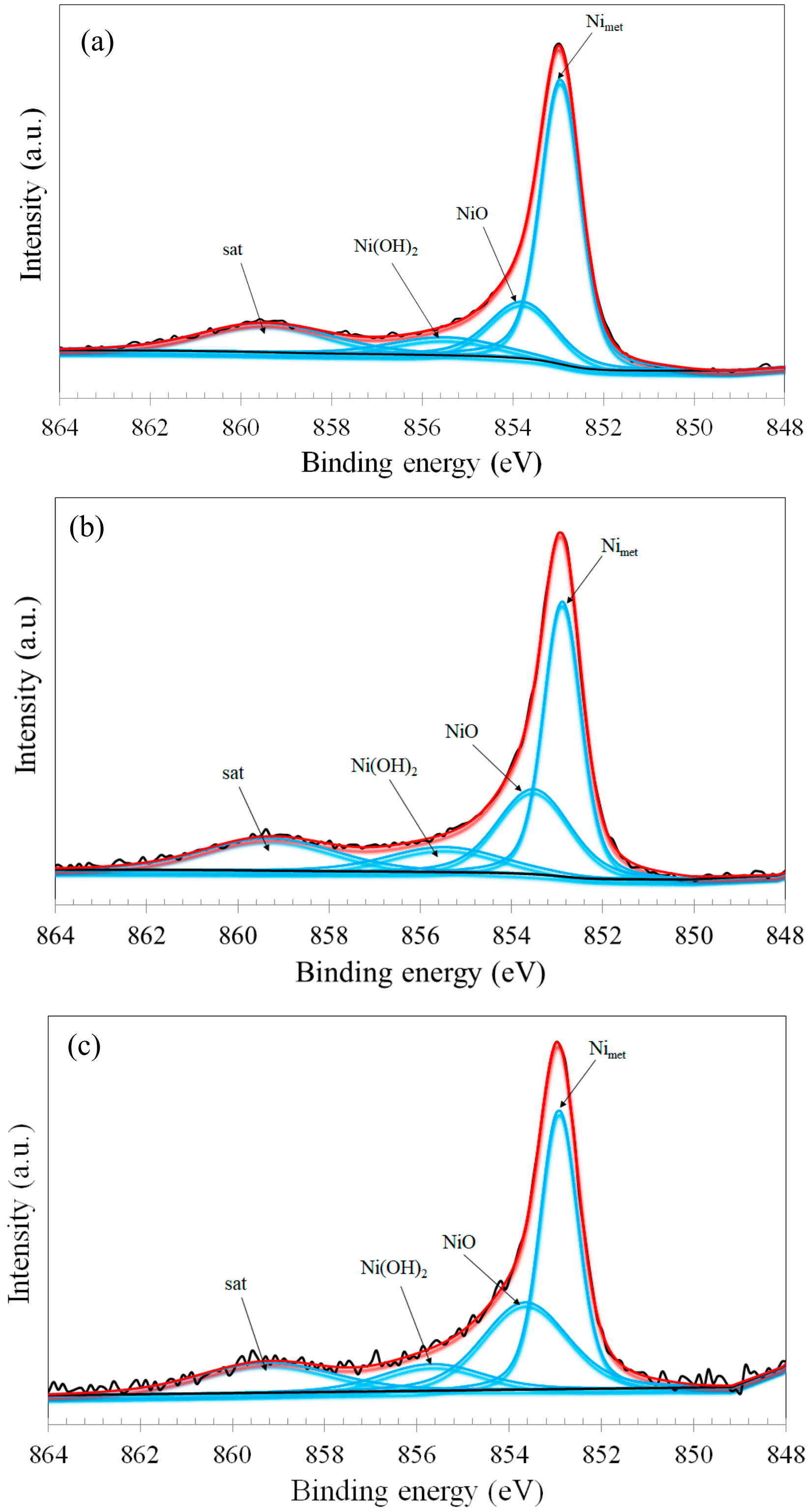

Figure S4. XPS narrow scan spectra in the Ni 2p$_{3/2}$ region: a) naturally formed passive film; b) wear track of the OCP sample; c) wear track of the -100 mV vs. OCP.

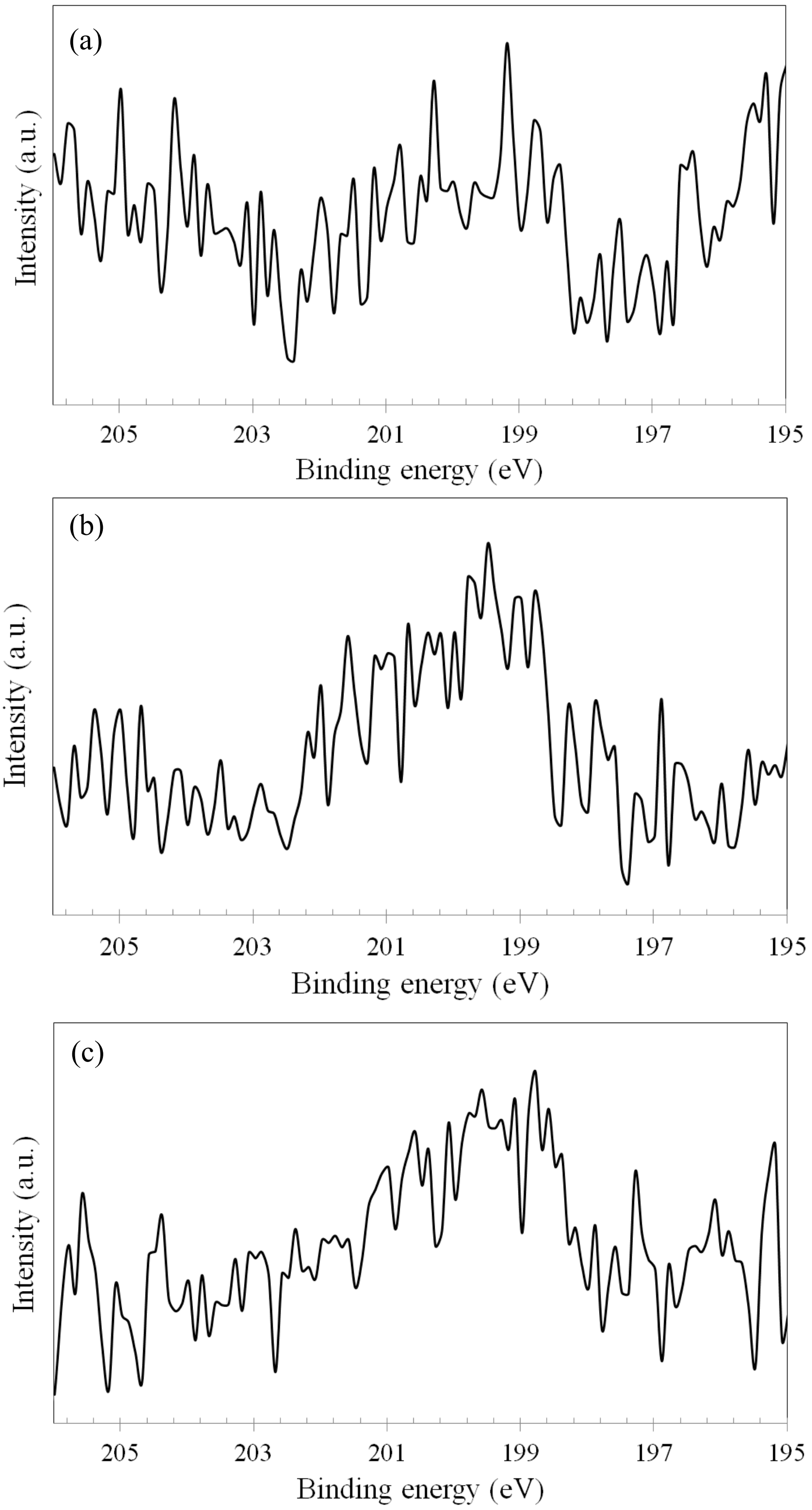

Figure S5. XPS narrow scan spectra in the Cl 2p region: a) +200 mV vs. Ag/AgCl; b) +700 mV vs. Ag/AgCl; c) -100 mV vs. OCP.
